# Associations of adolescent psychosocial factors to later benzodiazepine use: a population-based follow-up study of adolescent psychiatric inpatients in Northern Finland

**DOI:** 10.1097/YIC.0000000000000441

**Published:** 2022-11-11

**Authors:** Emmi Kujala, Helinä Hakko, Pirkko Riipinen, Kaisa Riala

**Affiliations:** a Faculty of Medicine, Research Unit of Clinical Medicine, Psychiatry, University of Oulu; b Oulu University Hospital, Psychiatry, Oulu, Finland

**Keywords:** adolescent, benzodiazepines, bullying, conduct disorder, substance-related disorders, suicide attempt

## Abstract

We investigated factors associated with benzodiazepine (BZD) use during late adolescence and early adulthood. The study population consisted of 508 adolescents admitted to psychiatric inpatient care between April 2001 and March 2006. Information on adolescents’ family- and school-related factors, suicidality and psychiatric disorders were obtained by semistructured interviews. Data on BZD prescriptions from 1999 to 2012 were collected from the Social Insurance Institution of Finland. In males heavy BZD use associated with adolescent substance-use disorder (OR, 3.5; *P* < 0.004) and parents’ psychiatric problems (OR, 3.5; *P* = 0.029). Among females, conduct disorder (OR, 3.3; *P* = 0.016), being a bully/bully-victim (OR, 3.3; *P* = 0.019) and parental substance-use problems (OR, 2.6; *P* = 0.024) were related to heavy BZD use. The mean (±SD) age of first BZD prescription was significantly lower in heavy, compared with mild users (men: 19.3 ± 2.5 vs. 21.0 ± 2.5 years, *P* = 0.027; women: 19.7 ± 2.6 vs. 21.5 ± 3.4 years, *P* = 0.027). Heavy, compared with mild, BZD use is associated with female suicide attempts (OR, 5.0; *P* = 0.049). Physicians should be cautious when prescribing BZDs to young adults and must allocate treatment to those with carefully evaluated clinical indications.

## Introduction

Benzodiazepines (BZDs) are a group of drugs that bind allosterically to specific receptors on gamma-aminobutyric acid chloride channel and potentiate the inhibitory effects of the channel. Consequently, BZDs reduce the amount of action potentials and turnover of several neurotransmitters (Lader, 2011). BZDs have relaxing and sedative effects and are mainly used to treat anxiety, sleeping disorders and epileptic seizures. BZDs are sometimes combined with other medical treatments for schizophrenia, depression, alcohol withdrawal and chronic pain (Kurtz, *et al.*, 2017).

The potential adverse effects of BZDs, such as daytime drowsiness, cognitive decline and deficiency in balance resulting falls, are well known (Webar and Duchemin, 2018). Together with opioids, BZDs are the most commonly abused prescription drugs (Substance Abuse Mental Health Services Administration, 2014). Therefore, long-term use of BZDs is not recommended in prescription guidelines (American Psychiatric Association 1998; NICE, 2011; Baldwin *et al.*, 2014). However, BZDs are still often (even one in four patients with anxiety disorder) prescribed for durations longer than 12 weeks (Tanguay Bernard *et al.*, 2018). Long-term use of BZDs increases the risk for dependence and cognitive and psychomotor impairment (Tanguay Bernard *et al.*, 2018; Vicens *et al.*, 2019). When prescribing BZDs to treat anxiety, long-acting agents should be preferred, the smallest possible efficient dosage should be used and for only a short period (Weber and Duchemin, 2018).

The aim of the present study is to investigate the relation of adolescence-related psychosocial factors (i.e. family- and school-related characteristics), suicidality, nonsuicidal self-injury (NSSI) and adolescent psychiatric disorders to participant’s prescribed BZDs during late adolescence or early adulthood. For this purpose, we utilized a population-based data of former adolescent psychiatric inpatients from Northern Finland. We focused on BZDs typically used to treat daytime anxiety (alprazolam, diazepam, oxazepam, lorazepam and chlordiazepoxide) and clonazepam, due to their potential risk for misuse, particularly among young adults (Kurtz *et al.*, 2017; Bouvier *et al.*, 2018). We also investigated whether the age of first BZD prescription differed between sexes and between mild and heavy BZD users.

## Methods

### Study population

This study is part of clinical follow-up project, which was initiated to examine the association of various psychosocial risk factors to the outcomes of severe psychiatric and substance-use disorders among adolescents. The initial study population consisted of 508 adolescents (13–17 years of age; boys: *n* = 208; 41% and girls: *n* = 300; 59%) who were admitted to the inpatient adolescent psychiatric ward at Oulu University Hospital between April 2001 and March 2006 (hereafter referred to as index hospitalization). The ward provides treatment for all adolescents from the area of Northern Finland (the provinces of Oulu and Lapland) requiring acute psychiatric hospitalization. The mean age of the study participants on admission was 15.5 years (SD = 1.3). The study was approved by the Ethics Committee of Oulu University hospital.

Regarding the central focus of our study, we excluded 63 study participants, because their BZD prescriptions were probably due to the presence of an epilepsy diagnosis (*n* = 19), or they had only a single or occasional prescriptions (*n* = 44) of BZDs during the follow-up. Thus, the final study population analyzed in this study comprised 445 former adolescent psychiatric inpatients (257 female and 188 male).

### Research instruments

The Schedule for Affective Disorders and Schizophrenia for School-Aged Children (K-SADS) to obtain Diagnostic and Statistical Manual of Mental Disorders, fourth edition (DSM-IV) diagnoses. K-SADS-PL is shown to be reliable method for defining DSM-IV psychiatric disorders in children and adolescents (Kaufman *et al.*, 1997; Ambrosini, 2000; Kim *et al.*, 2004). If any information based on the adolescent’s interview was deemed unreliable or missing, the parents or legal guardians were also interviewed using the K-SADS-PL framework. In addition, the European adaptation of the Addiction Severity Index (EuropASI), a semistructured interview (Kokkevi *et al.*, 1998), was used to gather information about the home environment at admission, parental characteristics and school-related factors.

### Register-based data on benzodiazepine purchases

Register-based information about physician-prescribed BZD purchases (The Anatomical Therapeutic Chemical code: N03AE01, N05BA01, N05BA02, N05BA04, N05BA06 and N05BA12) of the study participants was obtained from the Social Insurance Institution (SII) of Finland. The data regarding prescriptions were available up to the end of 2012 (SII, 2022) when the age of participants varied between 19 and 30 years. The age (in years) at first prescription of BZD, as well as the total number of prescriptions of BZDs, was calculated for each study participant.

We categorized the subjects with BZD prescriptions into two subgroups based on the amount and the total cost (in euros) of BZD purchases:

(1). ‘Mild users’ had nine or less prescriptions or the total amount of money spent on BZDs during the follow-up time was 50 euros or less.
(2). ‘Heavy users’ had either 9 or more prescriptions or had spent 50 euros or more on purchases.

The reason we took into consideration the money spent on BZDs, in addition to the amount of medicine acquired, was the differences in costs between different BZDs. The total number of BZD users in this study was 108 (*n* = 70 heavy users and *n* = 38 mild users). The control group (*n* = 337) comprised participants without any BZD purchases.

### Adolescence-related characteristics

#### Family environment

K-SADS-PL provided information on adolescents’ family structure and home environment before hospitalization. The adolescents were categorized into three different subgroups: (a) two-parent family (two biological parents or blended family), (b) single-parent family, and (c) out-of-home placement (child welfare placement, foster family, living alone or in a residential home).

#### Characteristics of the parents

Information on parental employment status (at least one parent had full/part-time work, yes/no) and problems related to parental mental health and substance-use was obtained from the EuropASI. Parental mental health and substance-use problems were based on each adolescent’s self-report as to whether they perceived that their mother or father would have required treatment for psychiatric (yes/no) or substance use (alcohol, drugs) problems (yes/no).

#### Bullying behavior

Information on bullying behavior was obtained from the K-SADS-PL. In the School Adaptation and Social Relations section of the interview, adolescents were asked whether they had ever been victims of bullying. Information on the bullying behavior was based on screening items regarding conduct disorder. The adolescents were asked: ‘Has there ever been a time when any kids really got on your nerves? Did you sometimes do things to get back at them (called names, threatened to beat up, pushed, tripped, knocked books out of one’s hand, slapped in the face)? How often did you do these things?’ Bullying was regarded as being present if the subjects had threatened or intimidated another on three or more occasions (Luukkonen *et al.*, 2011). The following three categories for bullying behavior was formed: (a) no involvement in bullying behavior, (b) victim of bullying, (c) bully/bully-victim (participants being bullies or both bullies and victims of bullying).

#### School-related factors

Factors related to school were also obtained from the K-SADS-PL interview. Each study participant was asked whether they had repeated a year/grade (yes vs. no) or received additional help (special education or placement in special class) at school (yes vs. no).

#### Suicidality and nonsuicidal self-injury

Information on an adolescent’s current suicidality (suicidal ideation, suicide attempts) and NSSI was based on the K-SADS-PL’s section on depressive disorder. Suicidal ideation was defined as being present when thoughts of suicide had been recurrent, and the adolescent had also considered the method of suicide. The information on suicide attempts, their seriousness and medical lethality was retrieved from two questions from the K-SADS-PL: (a) ‘have you actually tried to kill yourself?’, and (b) ‘how close were you to dying after your most serious suicidal act?’ The ‘suicide attempts’ group consisted of those adolescents who were at threshold level in either of the two items for suicidality. In terms of suicidality, study participants were categorized into three subgroups: (a) no suicidal behavior, (b) suicidal ideation and (c) an attempted suicide. Additionally, the NSSI was defined to be present (yes/no) if an adolescent had frequently carried out physical self-harming acts (>4 times a year).

#### Adolescent psychiatric disorders

The DSM-IV psychiatric disorders were assessed using the K-SADS-PL interview. These disorders were classified into five major psychiatric diagnostic groups, as follows: psychotic disorders (DSM-IV: 295, 296.0, 296.4–296.9, 297.1–297.3, 298.8–298.9, 301.13 and 301.22), anxiety disorders (300.00–300.02, 300.21–300.23, 300.29, 300.3, 308.3 and 309.81), affective disorders (296.2–296.3, 300.4 and 311), conduct disorders including ADHD (312.8–312.9, 313.81, 314 and 299.80) and substance-use disorders (303.9, 304.0–304.6, 304.8–304.9, 305.0, 305.2–305.7 and 305.9). An adolescent may have had diagnoses belonging to multiple categories of psychiatric disorders.

### Statistical methods

The statistical significance of group differences was assessed with Pearson’s Chi-square or Fisher’s Exact test. A logistic regression analysis was used to examine the association of psychiatric disorders, diagnosed in adolescence, to the likelihood of BZD use by early adulthood, after controlling for adolescence-related covariates (family type, parental characteristics, bullying behavior and school-related factors). When calculating the age at first BZD prescription, one single prescription of BZD for one study participant was left out from our analysis as it was deemed a potential outlier, because it was occurred at 4 years of age. The statistical software used in analyses was IBM SPSS statistics, version 27 (IBM Corp., Armonk, New York, USA).

## Results

In our total sample of former adolescent inpatients (*n* = 445), the use of BZDs was found in 108 (24.3%) study participants, the proportions being 53 (28.2%) male (*n* = 188) and 55 (21.4%) female (*n* = 257) participants. In male participants with BZDs, 16 (30.2%) were mild and 37 (69.8%) heavy users of BZDs, whereas in female participants 22 (40.0%) were mild and 33 (60.0%) heavy users of BZDs.

### Age of first benzodiazepine prescription

**Fig. 1:**
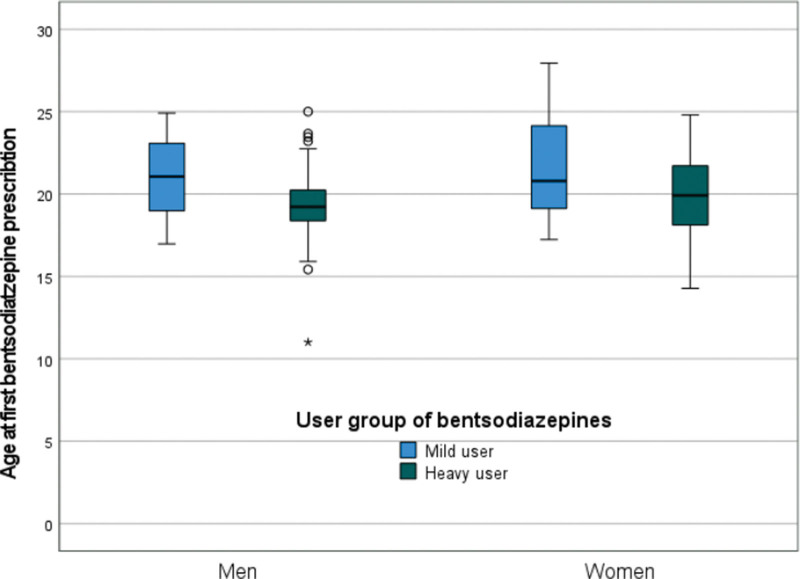
Age at first benzodiazepine prescription.

As Fig. 1 illustrates, the mean (±SD) age of first BZD prescription was statistically significantly lower in heavy compared with mild users of BZDs (male: 19.3 ± 2.5 years vs. 21.0 ± 2.5 years, *P* = 0.027; female: 19.7 ± 2.6 years vs. 21.5 ± 3.4 years, *P* = 0.027). There was no statistically significant sex difference in age of first BZD prescription either among mild (*P* = 0.905) or heavy BZD users (*P* = 568).

### Adolescence-related characteristics and use of benzodiazepines in men

Table 1 shows the adolescence-related characteristics of male study participants, by BZD user groups. The male BZD users, compared with controls, were characterized by higher age at admission to adolescent psychiatric inpatient care (16.1 vs. 15.1 years, *P* < 0.001), family structure being less commonly two-parent family (30.2% vs. 46.7%, *P* = 0.039) but more commonly out-of-home placement (52.8% vs. 31.9%; *P* = 0.008), parents with psychiatric problems (20.8% vs. 9.6%; *P* = 0.040), and they had substance-use disorder diagnosed during index hospitalization in adolescence (62.3% vs. 34.8%; *P* = 0.001). Analyses within male BZD users (Table 1) revealed that heavy users differed significantly from mild users only in a history of suicide attempts (8% vs. 31%; *P* = 0.031).

**Table 1:** Adolescence-related characteristics of the male study participants with and without use of benzodiazepines by young adulthood, and among heavy and mild users of benzodiazepines

Men	Study groups			Users of BZDs		
	BZD users (*n* = 53)	Controls (*n* = 135)	*P*-value	Heavy users (*n* = 37)	Mild users (*n* = 16)	*P*-value
Age at admission to adolescent psychiatric inpatient care, mean (SD)	16.1 (1.1)	15.1 (1.4)	<0.001	16.1 (1.2)	16.1 (1.0)	0.688
Family structure, *n* (%)						
Two-parent family	16 (30.2)	63 (46.7)	0.039	11 (29.7)	5 (31.3)	0.912
Single parent family	9 (17.0)	29 (21.5)	0.489	4 (10.8)	5 (31.3)	0.069
Out-of-home placement	28 (52.8)	43 (31.9)	0.008	22 (59.5)	6 (37.6)	0.142
Characteristics of parent(s), *n* (%)						
Parent(s) not at work	18 (34.0)	33 (24.4)	0.187	12 (32.4)	6 (37.5)	0.721
Psychiatric problems	11 (20.8)	13 (9.6)	0.040	8 (21.6)	3 (18.8)	0.813
Substance-use problems	17 (32.1)	38 (28.1)	0.594	14 (37.8)	3 (18.8)	0.172
Bullying behavior, *n* (%)						
No bullying behavior	25 (47.2)	56 (41.5)	0.479	17 (45.9)	8 (50.0)	0.786
Victim of bullying	12 (22.6)	45 (33.3)	0.151	7 (18.9)	5 (31.3)	0.325
Bully/bully-victim	16 (30.2)	34 (25.2)	0.485	13 (35.1)	3 (18.8)	0.233
Special services at school	33 (62.3)	84 (62.2)	0.996	25 (67.6)	8 (50.0)	0.226
Repeated grades	14 (26.4)	31 (23.0)	0.618	11 (29.7)	3 (18.8)	0.405
Suicidality						
No suicidal behavior	32 (60.4)	95 (70.4)	0.188	23 (62.2)	9 (56.3)	0.686
Only suicide ideation	13 (24.5)	27 (20.0)	0.495	11 (29.7)	2 (12.5)	0.181
Suicide attempts	8 (15.1)	13 (9.6)	0.285	3 (8.1)	5 (31.3)	0.031
Nonsuicidal self-injury (NSSI)	10 (18.9)	15 (11.1)	0.159	7 (18.9)	3 (18.8)	0.988
Adolescent psychiatric disorders						
Psychotic disorder	11 (20.8)	17 (12.6)	0.157	8 (21.6)	3 (18.8)	0.813
Anxiety disorder	6 (11.3)	21 (15.6)	0.456	6 (16.2)	0 (0.0)	0.087
Affective disorder	21 (39.6)	49 (36.3)	0.671	16 (43.2)	5 (31.3)	0.412
Conduct disorder	33 (62.3)	75 (55.6)	0.403	24 (64.9)	9 (56.3)	0.553
Substance-use disorder	33 (62.3)	47 (34.8)	0.001	26 (70.3)	7 (43.8)	0.067

BZD, benzodiazepine.

When all adolescence-related characteristics of male participants (presented in Table 1) were entered into the logistic regression analysis (Table 2), as significant predictors for use of BZDs, compared with controls, remained higher age at admission [odds ratio (OR), 1.7; *P* < 0.001], parental psychiatric problems (OR, 3.5; *P* = 0.016) and substance-use disorder in adolescence (OR, 2.5; *P* = 0.015). Similarly, heavy use of BZDs, compared with controls, was related to higher age at admission (OR, 1.6; *P* = 0.009), parental psychiatric problems (OR, 3.5; *P* = 0.029) and substance-use disorder in adolescence (OR, 3.5; *P* = 0.004). Further, a decreased likelihood for suicide attempts in adolescence was observed (OR, 0.19; *P* = 0.043) when comparing heavy BZD users with mild users. In comparison of mild users of BZD with controls (data not reported in Table 2), only a higher age at admission (OR, 2.0; 95% confidence interval, 1.20–3.40; *P* = 0.008) was associated with mild BZD use.

**Table 2:** Statistically significant adolescence-related predictors for use of benzodiazepines of male study participants

Men	OR (95% CI)	*P*-value
BZD users (*n* = 53) vs. controls (*n* = 135)		
Age at admission to adolescent psychiatric care	1.70 (1.26–2.29)	<0.001
Parental psychiatric problems	3.46 (1.26–9.53)	0.016
Substance-use disorder in adolescence	2.45 (1.19–5.05)	0.015
Heavy user of BZDs (*n* = 37) vs. controls (*n* = 135)		
Age at admission to adolescent psychiatric care	1.56 (1.12–2.17)	0.009
Parental psychiatric problems	3.52 (1.14–10.90)	0.029
Substance-use disorder in adolescence	3.47 (1.49–8.11)	0.004
Heavy (*n* = 37) vs. mild (*n* = 16) user of BZDs		
Suicide attempts	0.19 (0.40–0.95)	0.043

A logistic regression analysis (stepwise method with forward selection criteria).

Mild users of BZD with controls: higher age at admission (OR, 2.0; 95% CI, 1.20–3.40; *P* = 0.008) was associated to mild BZD use.

BZD, benzodiazepine; CI, confidence interval; OR, odds ratios.

### Adolescence-related characteristics and use of benzodiazepines in women

As seen in Table 3, the female participants using BZDs, compared with controls, were older at admission to adolescent inpatient care (15.9 vs. 15.4 years; *P* = 0.010), their family structure was less commonly two-parent family (45% vs. 57%; *P* = 0.046) but more commonly out-of-home placement (42% vs. 23%; *P* = 0.006), parents had substance-use problems (47% vs. 29%; *P* = 0.009), they less commonly belong to the group of no bullying behavior (33% vs. 52%; *P* = 0.011) but more commonly were bullies/bully-victims (33% vs. 7%; *P* < 0.001), they have received special services (60% vs. 37%; *P* = 0.002) and repeated grades (18% vs. 7%; *P* = 0.017) in school, and they were diagnosed with anxiety (40% vs. 26%; *P* = 0.047) and conduct (51% vs. 34%; *P* = 0.005) disorders during index hospitalization in adolescence. Within female BZD users, the heavy users differed from mild users with regard to bullying behavior (no bullying behavior, 21% vs. 50%, *P* = 0.026; bullies/bully-victims, 46% vs. 14%, *P* = 0.014), previous suicide attempts (39% vs. 14%; *P* = 0.039), and diagnosis for conduct (67% vs. 27%; *P* = 0.004) and substance-use (64% vs. 23%; *P* = 0.003) disorder.

**Table 3:** Adolescence-related characteristic of the female study participants with and without use of benzodiazepines by young adulthood, and among heavy and mild users of benzodiazepines

Women	Study groups			Users of BZDs		
	BZD users (*n* = 55)	Controls (*n* = 202)	*P*-value	Heavy users (*n* = 33)	Mild users (*n* = 22)	*P*-value
Age at admission to adolescent psychiatric care, mean (SD)	15.9 (1.2)	15,4 (1.3)	0.010	16.0 (1.2)	15.7 (1.2)	0.480
Family structure, *n* (%)						
Two-parent family	25 (45.4)	115 (56.9)	0.046	13 (39.4)	10 (45.5)	0.655
Single parent family	9 (16.4)	40 (19.8)	0.565	4 (12.1)	5 (22.7)	0.298
Out-of-home placement	23 (41.8)	47 (23.3)	0.006	16 (48.5)	7 (31.8)	0.220
Characteristics of parent(s), *n* (%)						
Parent(s) not at work	12 (21.8)	38 (18.8)	0.618	8 (24.2)	4 (18.2)	0.594
Psychiatric problems	17 (30.9)	40 (19.8)	0.079	10 (30.3)	7 (31.8)	0.905
Substance-use problems	26 (47.3)	58 (28.7)	0.009	17 (51.5)	9 (40.9)	0.440
Bullying behavior, *n* (%)						
No bullying behavior	18 (32.7)	105 (52.0)	0.011	7 (21.2)	11 (50.0)	0.026
Victim of bullying	19 (34.5)	76 (37.6)	0.675	11 (33.3)	8 (36.4)	0.817
Bully/bully-victim	18 (32.7)	21 (10.4)	<0.001	15 (45.5)	3 (13.6)	0.014
Special services at school	33 (60.0)	74 (36.6)	0.002	21 (63.6)	12 (54.5)	0.500
Repeated grades at school	10 (18.2)	15 (7.4)	0.017	7 (21.2)	3 (13.6)	0.475
Suicidal behavior						
No suicidal behavior	22 (40.0)	78 (38,6)	0.852	12 (36,4)	10 (45,5)	0.500
Only suicide ideation	17 (30.9)	72 (35.6)	0.513	8 (24.2)	9 (40.9)	0.190
Suicide attempts	16 (29.1)	52 (25.7)	0.618	13 (39.4)	3 (13.6)	0.039
Nonsuicidal self-injury (NSSI)	22 (40.0)	81 (40.1)	0.989	16 (48.5)	6 (27.3)	0.116
Adolescent psychiatric disorders						
Psychotic disorder	9 (16.4)	25 (12.4)	0.439	3 (9.1)	6 (27.3)	0.074
Anxiety disorder	22 (40.0)	53 (26.2)	0.047	14 (42.4)	8 (36.4)	0.653
Affective disorder	31 (56.4)	121 (59.9)	0.636	19 (57.6)	12 (54.5)	0.824
Conduct disorder	28 (50.9)	62 (30.7)	0.005	22 (66.7)	6 (27.3)	0.004
Substance-use disorder	26 (47.3)	68 (33.7)	0.063	21 (63.6)	5 (22.7)	0.003

BZD, benzodiazepine.

In the assessment of statistically significant adolescence-related predictors for use of BZDs among female participants (Table 4), the significant predictors for use of BZDs, compared with controls, were shown to be higher age at admission (OR, 1.5; *P* = 0.008), parental substance-use problems (OR, 1.9; *P* = 0.048), being a bully/bully-victim (OR, 3.0; *P* = 0.005) and having had special services in school (OR, 2.1; *P* = 0.033). Further, heavy use of BZDs in female participants, compared with controls, was related to higher age at admission (OR, 1.7; *P* = 0.006), parental substance-use problems (OR, 2.6; *P* = 0.024), being bully/bully-victim (OR, 3.3; *P* = 0.019) and being diagnosed with conduct disorder during index hospitalization in adolescence (OR, 3.3; *P* = 0.016). In comparison between heavy users and mild users, significant predictors for heavy use of BZDs were suicide attempts (OR, 5.00; *P* = 0.049), and a diagnosis of conduct (OR, 5.0; *P* = 0.019) and substance-use (OR, 4.5; *P* = 0.029) disorder in adolescence. None of the adolescence-related characteristics in females were associated with mild use of BZDs, compared with controls.

**Table 4:** Statistically significant adolescence-related predictors for use of benzodiazepines of female study participants

Women	OR (95% CI)	*P*-value
BZD users (*n* = 55) vs. controls (*n* = 202)		
Age at admission to adolescent psychiatric care	1.46 (1.10–1.92)	0.008
Parental substance-use problems	1.93 (1.00–3.71)	0.048
Bully/bully-victims	3.01 (1.38–6.55)	0.005
Special services in school	2.06 (1.06–4.01)	0.033
Heavy BZD user (*n* = 33) vs. controls (*n* = 202)		
Age at admission to adolescent psychiatric care	1.67 (1.16–2.40)	0.006
Parental substance-use problems	2.61 (1.13–6.00)	0.024
Bully/bully-victims	3.30 (1.21–9.00)	0.019
Conduct disorder in adolescence	3.33 (1.25–8.82)	0.016
Heavy (*n* = 33) vs. mild (*n* = 22) user of BZDs		
Suicide attempts	4.97 (1.01–24.51)	0.049
Conduct disorder in adolescence	5.00 (1.31–19.14)	0.019
Substance-use disorder in adolescence	4.46 (1.16–17.10)	0.029

A logistic regression analysis (stepwise method with forward selection criteria).

None of the adolescence-related characteristics were associated with mild use of BZDs compared to controls.

BZD, benzodiazepine; CI, confidence interval; OR, odds ratios.

## Discussion

Our study of former adolescent psychiatric inpatients showed that adolescent substance-use disorder, in both sexes, and conduct disorder among females, was related to heavy BZD use later in life. Suicide attempts in adolescence were associated with heavy BZD use in females but, interestingly, heavy BZD use was not associated with anxiety-, mood- or psychotic disorders, where short-term BZD use may be clinically justified.

Our study showed that the younger the age at first BZD prescription, the more likely it was a participant became a heavy BZD user, both in male and female subjects. This finding is in line with earlier studies (Chen *et al.*, 2009). The study of Hammond *et al.* (2014) reported that the neurobiological basis for earlier age at onset of substance-use is associated with increased addiction severity and poorer outcomes among adolescents. We consider our finding important, as our study investigated BZD prescriptions, which should always be based on a physician’s careful clinical assessment of the patient receiving the medication. A notably high proportion of our study participants with heavy BZD use (70% of male and 64% of female participants) were diagnosed with substance-use disorder already during their index hospitalization, between the ages of 13–17 years. It is well known that BZD misuse is common among patients with substance misuse, along with misuse of opioids (Stein *et al.*, 2016; Votaw *et al.*, 2019), cocaine (Roy *et al.*, 2018) and alcohol (Votaw *et al.*, 2019). Further, BZDs are indicated to treat the symptoms of alcohol withdrawal (Bahji *et al.*, 2022). Our finding suggests that some of our study participants with a diagnosis of substance-use disorder in adolescence, may have misused BZDs repeatedly through frequent BZD prescriptions, without a valid clinical indication. Therefore, the potential for BZD misuse in this patient group should be considered critically when prescribing medications for any comorbid psychiatric disorders.

In the female study participants, bully or bully-victim status and conduct disorder were associated with later BZD use. In addition, the need for special services at school and repeated grades among females with later BZD use was higher than in the control group in our study. Behavioral problems, which are specified as conduct disorder, are usually associated with bullying behavior (Golmaryami *et al.*, 2016) and bullies are at risk of externalizing disorders, criminality and illicit drug misuse (Klomek *et al.*, 2015). Azevedo Da Silva and Martins (2020) reported that bullying perpetration was associated with alcohol, cannabis and cigarette use among youths. However, their study did not analyze prescription drug misuse, and, thus, we consider our finding of heavy BZD prescription use in bullies/bully-victims to be an important addition to the previous literature. Additionally, adolescents with conduct disorder may develop antisocial behavior, lasting up into adulthood. Females with antisocial behavior are commonly reported to suffer from various emotional problems (Pajer *et al.*, 2006). Adolescent girls with conduct disorder may also be traumatized when spending time with antisocial peers (Bernhard *et al.*, 2018), which may explain why they seek BZD prescriptions to treat their trauma-related anxiety.

In female, but not in male, study participants, adolescence-related suicide attempts were associated with heavy BZD use by young adulthood. As an earlier study has documented, women, compared with men, more commonly attempt suicide, particularly those with nonviolent methods, and they are more likely to report their suicidal behavior (Schrijvers *et al.*, 2012). One plausible explanation behind the sex difference in suicide attempts may be borderline personality disorder (BPD), which is predominantly diagnosed in women (Schulte Holthausen and Habel, 2018). Female study participants with suicide attempts in adolescence may have suffered from developing BPD and, consequently, seek BZD prescriptions to treat their anxiety linked to this underlying, still undiagnosed disorder. It is worth noting, however, that the number of male study participants reporting suicide attempts was relatively low, which warrants further studies, using larger databases, to explore whether male adolescents with suicidal behavior are less likely to receive BZD prescriptions, compared with their female peers.

In our study, parental psychiatric problems were shown to increase the likelihood of heavy BZD use among male study participants by over three-fold. In female subjects, parental substance-use problems more than doubled the likelihood of heavy BZD use. Our findings are in line with earlier studies, reporting that parental substance-use is associated with an increased likelihood for adolescents’ mental health problems and substance-use (Smith and Wilson, 2016; Vaughan *et al.*, 2017). Further, our findings of highly prevalent adolescent conduct disorder (males 65%, females 67%) and out-of-home living arrangements (males 59% and females 48%) among participants with heavy BZD use, suggest that multiproblematic living circumstances may lie behind early-onset heavy BZD use.

Interestingly, as our results of logistic regression analyses showed, neither adolescence-related anxiety, affective and psychotic disorders nor suicidal ideation or NSSI were associated with BZD use among the study participants. Using BZDs to temporarily treat anxiety is clinically justified in conditions where anxiety is the main symptom. In the Canadian study by Tanguay Bernard *et al.* (2018), a tendency for long-term BZD use was found among adults with anxiety disorders. Earlier literature suggests that BZD prescriptions are frequently (55%) written by primary care physicians rather than psychiatric specialists (Cascade and Kalali, 2008). In Finland, there is a care guarantee for adolescent specialized level psychiatric care. Patients below the age of 23 and in need of psychiatric investigation must be evaluated in a special health care unit within 6 weeks of the initial referral, and treatment should be instigated within 3 months (EU-heathcare.fi). Consequently, our study participants were likely to maintain their after-care contact with a psychiatric specialist after their adolescent hospitalization between the ages of 13–17 years. Therefore, one explanation for the absence of heavy use of BZDs of study participants with internalizing disorders in adolescence is the continuity of care they have received through contacts in outpatient care with psychiatric specialists. These clinicians were probably more cautious when prescribing BZDs and more likely to offer alternative psychosocial treatments.

### Limitations and strengths

A strength of our study is that psychiatric disorders and the background information on family- and school-related factors in adolescence were based on information from the valid semistructured K-SADS-PL – interview and EuropASI – questionnaire completed by trained medical professionals. Furthermore, in Finland, all permanent residents are entitled to reimbursements for prescription medications provided by the SII of Finland (SII, 2022) and, therefore, all BZD purchases as prescription medication are captured from records of SII. However, we were not able to calculate the defined-daily-doses, as our data did not include information on the strengths or different package sizes of BZDs. An important observation is that our results are likely to underestimate the true levels BZD use, because medicines may also have been illegally purchased on the black market. Our study population represents adolescents in need of psychiatric inpatient treatment and, thus, our findings cannot directly be generalized to the whole Finnish adolescent population. We focused on adolescence-related factors in terms of later use of BZDs, which evidently calls for further research to examine these associations in terms of psychiatric morbidity after adolescent index hospitalization.

### Conclusion

Our findings suggest that BZD prescriptions among adolescents and young adults may not always be based on an adequate psychiatric assessment and appropriate clinical indications. In particular, the high levels of BZD use among participants with a history of adolescent substance-use disorder are an alarming finding. Adolescents with suspected substance-use problems need careful assessment, case management and age-specific treatment arranged in appropriate substance-use or other psychiatric services targeted to adolescents. A treatment plan should be devised cooperatively, with parents and/or significant others involved in all stages of an adolescent’s treatment program. Clinicians should be cautious when treating adolescents and young adults who are repeatedly seeking BZD prescriptions and should have a lower threshold for making referrals for diagnosis-based psychosocial interventions, including substance-use treatment.

## Acknowledgements

### Conflicts of interest

There are no conflicts of interest.
